# Protective Effects of *Achyranthes bidentata* Root Extract Against Benign Prostatic Hyperplasia Induced by Testosterone Propionate in Rats

**DOI:** 10.3390/nu18213573

**Published:** 2026-10-29

**Authors:** Han Kyul Kim, Phuong Tran, Sunmin Kim, Jungbin Song

**Affiliations:** 1 Graduate School, Kyung Hee University, 26 Kyungheedae-ro, Dongdaemun-gu, Seoul 02447, Republic of Korea; ruf213@naver.com (H.K.K.); mposkim@naver.com (S.K.); 2 Department of Herbal Pharmacology, College of Korean Medicine, Kyung Hee University, 26 Kyungheedae-ro, Dongdaemun-gu, Seoul 02447, Republic of Korea; phuongtran24288@gmail.com

**Keywords:** *Achyranthes bidentata*, testosterone propionate, benign prostatic hyperplasia, dihydrotestosterone, androgen receptor, apoptosis

## Abstract

**Background/Objectives**: Benign prostatic hyperplasia (BPH) is prevalent among older men and is often accompanied by lower urinary tract symptoms (LUTS), including storage and voiding symptoms. Achyranthis Radix, the root of *Achyranthes bidentata*, is commonly used as a functional food in Asia and has been documented in traditional medicine for conditions associated with LUTS. However, its effects on BPH have not been experimentally investigated. Therefore, this study evaluated an ethanolic extract of Achyranthis Radix (ARE) in a rat model of testosterone propionate (TP)-induced BPH. **Methods**: Male Sprague-Dawley rats were randomly assigned to six groups: sham, control, finasteride (1 mg/kg), and ARE (30, 100, or 300 mg/kg). BPH was induced in all groups except the sham group by castration followed by subcutaneous administration of TP (3 mg/kg/day) for 4 weeks. ARE and finasteride were orally administered once daily concurrently with TP treatment. Prostate weight, prostate index, histological changes, serum androgen levels, androgen receptor (AR) expression, and apoptosis-related proteins were evaluated. **Results**: ARE significantly reduced prostate weight and prostate index and attenuated BPH-associated histological changes compared with the control group. ARE also significantly decreased serum dihydrotestosterone levels and reduced prostatic AR expression. In addition, ARE modulated the expression of apoptosis-related proteins in the prostate. **Conclusions**: ARE attenuated the development of prostate enlargement in rats when administered concurrently with TP. The accompanying changes in serum androgen levels and prostatic androgen-related and apoptosis-related protein expression provide a basis for further mechanistic investigation. These findings support further evaluation of ARE for BPH management.

## 1. Introduction

Benign prostatic hyperplasia (BPH) is a prevalent urological disorder whose histological features include the proliferation of smooth muscle and epithelial cells within the transition zone of the prostate [1]. The prevalence of BPH increases with age, with approximately 50% of men aged 51–60 years and more than 80% of men over 80 years of age showing histological evidence of BPH [2]. The global increase in life expectancy has resulted in a corresponding rise in the prevalence of BPH worldwide [3]. Prostatic enlargement may contribute to urethral compression and bladder outlet obstruction, leading to lower urinary tract symptoms (LUTS), which can be classified into storage symptoms (frequent urination, urgency, and nocturia) and voiding symptoms (hesitancy, straining, slow stream, intermittency, and terminal dribbling). Although LUTS associated with BPH are rarely life-threatening in elderly men, these symptoms can significantly affect their quality of life [4].

Despite the unclear etiology of BPH, age-related androgen dysregulation is known to play a major role in mediating the pathogenesis of BPH [5,6]. Prostate growth is largely dependent on androgen signaling, particularly that mediated by testosterone and dihydrotestosterone (DHT). Upon androgen binding, the androgen receptor (AR) translocates to the nucleus and, as a ligand-dependent transcription factor, activates androgen-responsive signaling involved in prostate cell growth, thereby contributing to the development of BPH [7].

Pharmacological therapies for BPH include alpha-1 blockers and 5-alpha reductase inhibitors. Alpha-1 blockers improve urinary flow by reducing smooth muscle tone in the prostate and bladder neck, but do not prevent the progression of BPH [8]. Tamsulosin, the most commonly prescribed alpha-1 blocker, has been reported to cause side effects such as dizziness and retrograde ejaculation, occurring in 10–12% of cases [9]. Finasteride and dutasteride inhibit 5-alpha reductase, thereby lowering DHT production and reducing prostate size. Sexual adverse effects, such as reduced libido and erectile dysfunction, have been reported in 3.4–15.8% of patients receiving these drugs [10]. Concerns regarding these adverse effects, particularly sexual dysfunction, have contributed to growing interest in alternative approaches for the management of BPH. In Europe, in particular, previous studies have shown that up to 50% of BPH prescriptions are herbal and alternative medicines [11].

*Achyranthes bidentata* Blume is a perennial plant in the Amaranthaceae family and is widely used in Asia as a functional food and herbal medicine [12]. In East Asian countries, including Korea, China, and Japan, its roots, known as Achyranthis Radix, have been used in traditional medicine to manage conditions such as arthritis, amenorrhea, lumbago, stranguria, and headache [13,14]. Extensive pharmacological research on Achyranthis Radix has revealed a range of activities, including anti-osteoporosis [15,16], anti-osteoarthritis [17,18], neuroprotective [19], and immunomodulatory effects [20]. The major bioactive components identified in Achyranthis Radix include oleanane-type triterpenoid saponins, phytoecdysteroids, polysaccharides, and polypeptides [21].

Achyranthis Radix has been documented for its traditional use in addressing urinary incontinence [22], one of the LUTS that can negatively affect quality of life in older adults [23]. Furthermore, Achyranthis Radix has been used as a constituent herb in multi-herbal prescriptions for the treatment of BPH in traditional Asian medicine [24,25,26]. These traditional uses suggest the potential relevance of Achyranthis Radix to BPH; however, its effects on BPH have not been experimentally investigated. Therefore, the present study aimed to evaluate the effects of Achyranthis Radix in a testosterone propionate (TP)-induced BPH rat model and examine associated hormonal and protein expression changes.

## 2. Materials and Methods

### 2.1. Preparation of Achyranthis Radix Extract (ARE)

Dried roots of *A. bidentata* (Henan Province, China) were purchased from Dongwoodang Pharmacy Co., Ltd. (Yeongcheon, Republic of Korea), and authenticated by Prof. Jungbin Song of Kyung Hee University. The voucher specimen was deposited in the Herbarium of the Department of Herbal Pharmacology, College of Korean Medicine, Kyung Hee University (No. 21081801). The roots were ground and extracted under reflux conditions with 70% ethanol (solid-to-liquid ratio of 1:10) for 3 h at 80 °C. The extract was filtered using Whatman™ Grade 2 qualitative filter papers (Cytiva, Marlborough, MA, USA). Following rotary evaporation at 45 °C under reduced pressure, the concentrate was lyophilized using a freeze dryer (FDU-1200, EYELA, Tokyo, Japan) to yield a powder (extraction yield, 50.9%).

### 2.2. High-Performance Liquid Chromatography (HPLC) Analysis

For quantitative quality control of ARE, the β-ecdysterone content was determined by HPLC analysis. β-Ecdysterone was selected as an analytical marker based on previous phytochemical research [27] and its use in the Chinese Pharmacopoeia (2020 ed.), which specifies a minimum β-ecdysterone content of 0.030% (*w*/*w*), calculated on a dried basis, in Achyranthis Bidentatae Radix [28]. A reference standard of β-ecdysterone was obtained from Merck (Darmstadt, Germany). For analysis, 100 mg of ARE was mixed with 10 mL of 70% methanol in deionized water (*v*/*v*) and sonicated for 15 min at 25 °C. After sonication, the solution was filtered through a 0.45 µm syringe filter, and 20 µL was injected into the HPLC system. An Agilent 1260 HPLC system (Agilent Technologies, Santa Clara, CA, USA) equipped with a quaternary pump (G7111A), a diode array detector (G7155A), and an autosampler (G7129A) was used. The marker compound was separated on an Agilent Zorbax Eclipse Plus C18 column (4.6 × 250 mm i.d., 5 µm particle size) maintained at 40 °C using an isocratic mobile phase of acetonitrile–water–formic acid (16:84:0.1, *v*/*v*/*v*). The flow rate was 1 mL/min, and the detection wavelength was set at 250 nm. Each analysis was performed in triplicate.

### 2.3. Animals

Male Sprague-Dawley rats, aged 10 weeks, were purchased from Koatech (Pyeongtaek, Republic of Korea) and were used in this study according to the guidelines of the Institutional Animal Care and Use Committee of Kyung Hee University (Approval No. KHSASP-23-130). Rats were housed in polycarbonate cages in a temperature-controlled room at 23 ± 1 °C with a humidity of 55–60% and a 12 h light–dark cycle. Rats were provided standard chow and had ad libitum access to distilled water.

### 2.4. TP-Induced BPH Rat Model

**Figure 1:**
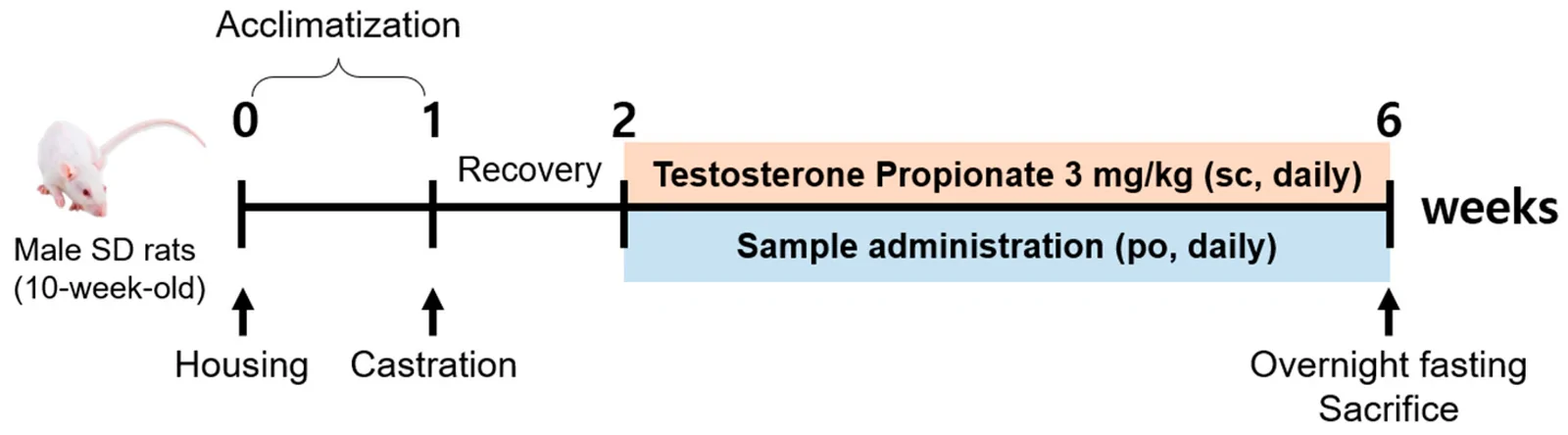
Experimental scheme.

After 1 week of acclimatization, rats were randomly divided into six groups with comparable baseline body weights: sham (*n* = 9), control (*n* = 10), finasteride 1 mg/kg (*n* = 10), ARE 30 mg/kg (*n* = 9), ARE 100 mg/kg (*n* = 9), and ARE 300 mg/kg (*n* = 9). The induction of BPH and sample administration were conducted as depicted in Figure 1. Bilateral orchiectomy was performed under isoflurane anesthesia in all rats other than those in the sham group, according to the method reported previously [29]. After a midline abdominal incision, the testes, epididymides, and epididymal fat pads were removed, and the incision was sutured. The sham group underwent the same surgical procedure without removal of any tissues. Rats were maintained for 1 week under standard conditions after surgery to recover.

To induce BPH, orchiectomized rats were injected subcutaneously with TP (TCI, Tokyo, Japan) at a dose of 3 mg/kg/day (dissolved in olive oil) for 28 consecutive days. The sham group was injected with olive oil according to the same regimen. Both ARE and finasteride (Proscar®, MSD, Seoul, Republic of Korea) were administered orally once daily at a dosing volume of 10 mL/kg for 28 days concurrently with TP administration, while the sham and BPH control groups received distilled water at the same dosing volume and according to the same regimen.

ARE doses were expressed as milligrams of lyophilized extract powder per kilogram of body weight per day. The doses of 30, 100, and 300 mg/kg/day were used to explore dose-related effects across a tenfold range, with approximately threefold intervals between dose levels. Using body surface area-based conversion (*K*_m_ = 6 for rats and 37 for adult humans) [30], the highest dose of 300 mg/kg/day corresponds to approximately 2.92 g/day of extract for a 60-kg adult. Based on the extraction yield of 50.9%, this is equivalent to approximately 5.73 g/day of crude Achyranthis Radix, which falls within the traditional daily dosage of 5–12 g specified in the Chinese Pharmacopoeia (2020 ed.) [28]. The lower doses of 30 and 100 mg/kg/day were included to explore responses below this dose level.

At the end of the experiment, rats were fasted overnight and deeply anesthetized with isoflurane. Blood samples were collected from the inferior vena cava, and the rats were euthanized by exsanguination under deep anesthesia. The ventral, dorsolateral, and anterior prostate lobes were immediately excised and weighed. The prostate index was calculated as prostate weight (mg)/fasting body weight (g) × 100 and expressed as mg/100 g body weight. Ventral prostate tissues for histological assessment were fixed in 10% formalin, whereas samples for Western blot analysis were stored at −80 °C after snap-freezing in liquid nitrogen.

### 2.5. Histology

Following paraffin embedding, the fixed ventral prostate tissues were sectioned at a thickness of 4 μm. The sections were then stained with Harris’ hematoxylin and eosin (H&E), and examined under a light microscope (Eclipse Ci-L, Nikon, Tokyo, Japan). 

Histological changes were evaluated using a scoring system based on previously established methods [31,32]. The scoring system assessed histopathological features including luminal and acinar shape, interacinar space, stroma, epithelial shape, piling up and budding out of epithelial cells, lesion distribution, and nuclear shape and size. Higher scores indicated more severe histological changes. For each rat, five randomly selected fields were examined at 100× magnification and five at 400× magnification. All slides were independently evaluated by two investigators who were blinded to group allocation and applied the same predefined scoring criteria. The mean of the total scores assigned by the two investigators was used for statistical analysis.

Epithelial thickness was measured at 100× magnification across five randomly selected fields for each rat using ImageJ software (version 1.54h, NIH, Bethesda, MD, USA), and the mean value was calculated. Imaging and measurement of epithelial thickness were performed in a blinded manner with respect to group allocation.

### 2.6. Measurement of Serum Testosterone and DHT Concentrations

For serum separation, blood samples were left at room temperature for 60 min and then centrifuged at 4000 rpm at 4 °C for 10 min. The serum was subsequently stored at −80 °C until use. Serum testosterone and DHT concentrations were measured using competitive inhibition enzyme-linked immunosorbent assay kits (Cat. No. CSB-E05100r and CSB-E07879r, Cusabio Technology, Houston, TX, USA). The assays were performed according to the manufacturer’s instructions.

### 2.7. Western Blot Analysis

Four biologically independent animals from each of the sham, control, finasteride, and ARE 300 mg/kg groups were randomly selected for Western blot analysis. Animal selection was completed before quantitative analysis of the Western blots. Among the ARE groups, the 300 mg/kg group was selected because it showed the greatest numerical inhibition of the TP-induced increase in prostate index, the primary outcome measure in this study. Ventral prostate tissues were lysed using RIPA buffer (Thermo Scientific, Waltham, MA, USA) with a bead mill homogenizer (Bead Ruptor Elite, OMNI International, Kennesaw, GA, USA). The lysates were then centrifuged at 13,000 rpm for 10 min at 4 °C, and the resulting supernatants were collected into microcentrifuge tubes. Protein concentrations in the supernatants were quantified using the Pierce™ BCA Protein Assay (Thermo Scientific, Waltham, MA, USA). For SDS-PAGE, protein samples were mixed with 2× Laemmli buffer (Bio-Rad Laboratories, Hercules, CA, USA). Proteins (60 µg) were separated on 4–20% Mini-PROTEAN® TGX™ precast gels (Bio-Rad Laboratories) at 200 V for 30 min and then transferred to PVDF membranes (Bio-Rad Laboratories) at 100 V for 60 min. Membranes were blocked for 1 h at room temperature in TBST containing 5% skim milk. Primary antibody incubation was performed overnight at 4 °C using antibodies against AR (1:200), Bcl-2 (1:1000), Bax (1:1000), and β-actin (1:500) (Santa Cruz Biotechnology, Dallas, TX, USA). The membranes were then washed four times with TBST for 5 min per wash. For secondary antibody incubation, membranes were incubated for 1 h at room temperature with goat anti-mouse IgG (H+L) secondary antibodies (Invitrogen, Waltham, MA, USA), diluted to 1:5000 for β-actin and Bcl-2 and to 1:2000 for AR and Bax. After four additional 5-min washes with TBST, protein bands were detected using WesternBright ECL HRP substrate (Advansta, San Jose, CA, USA) and imaged with a ChemiDoc MP Imaging System (Bio-Rad Laboratories, Hercules, CA, USA). Band intensities were quantified using Image Lab Software (version 6.1, Bio-Rad Laboratories, Hercules, CA, USA), and target protein levels were normalized to the β-actin signal.

### 2.8. Statistical Analysis

Differences among groups were analyzed using one-way analysis of variance (ANOVA) followed by Dunnett’s multiple comparisons test using GraphPad Prism software (version 8.4.2, GraphPad Software, Inc., San Diego, CA, USA). A *p*-value < 0.05 was considered statistically significant. All values are expressed as the mean ± SD.

Effect sizes for prostate index, the primary outcome measure in this study, were estimated as Hedges’ *g* [33] with two-sided 95% confidence intervals for each ARE dose versus the control group, following ARRIVE 2.0 recommendations [34]. Effect-size analyses were conducted using the effectsize package (version 1.0.3) [35] in R (version 4.6.1; R Foundation for Statistical Computing, Vienna, Austria).

## 3. Results

### 3.1. HPLC Analysis of β-Ecdysterone in the ARE

**Figure 2:**
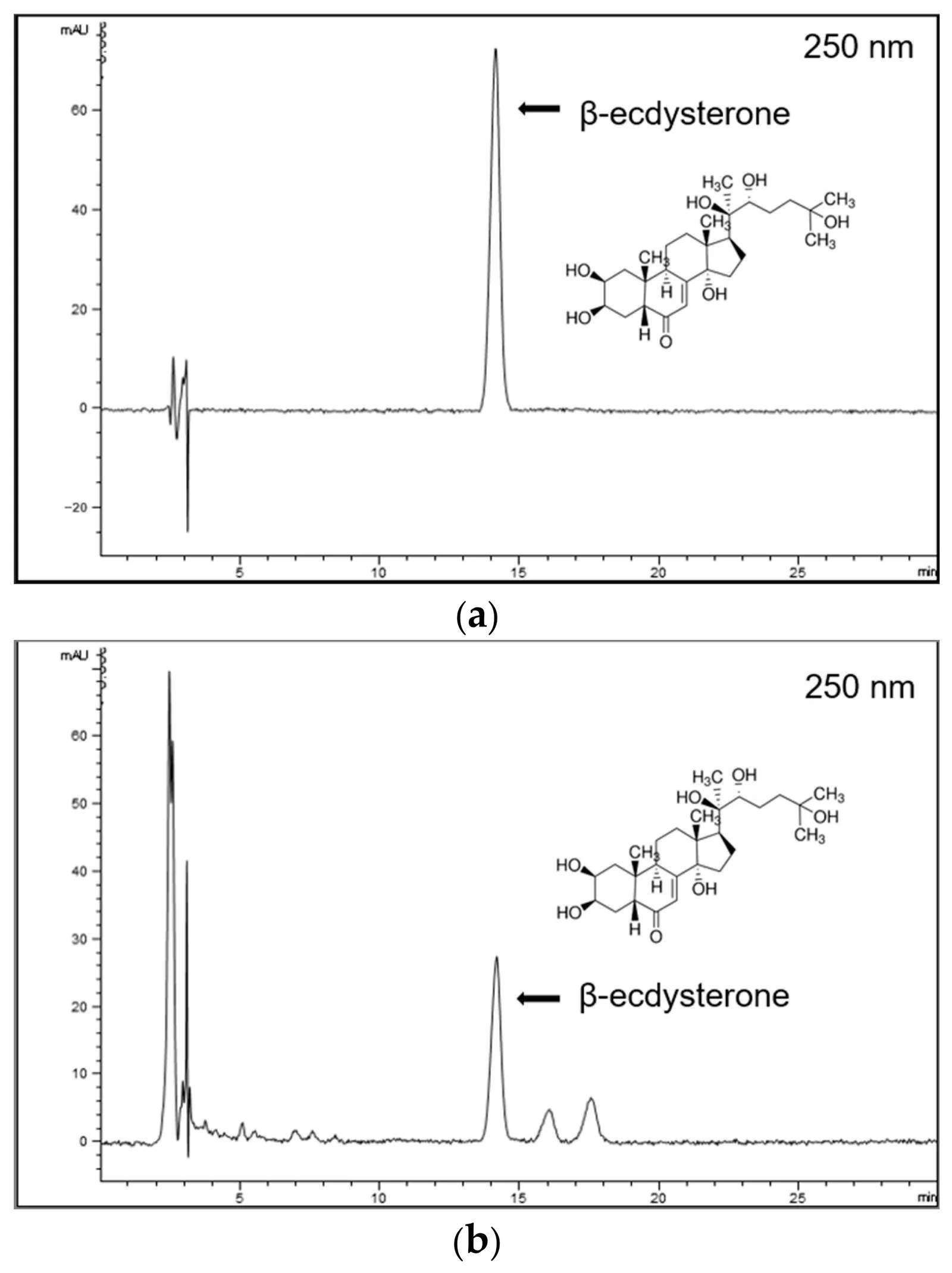
High-performance liquid chromatography chromatograms of the β-ecdysterone standard (**a**) and Achyranthis Radix extract (**b**).

The content of β-ecdysterone in the ARE powder was quantified using HPLC analysis. The representative chromatograms of the β-ecdysterone standard and ARE are shown in Figure 2a,b, respectively. β-Ecdysterone was detected at a retention time of 14.1 min and quantified as 1.88 mg/g of extract powder. 

### 3.2. Body Weight Changes 

**Figure 3:**
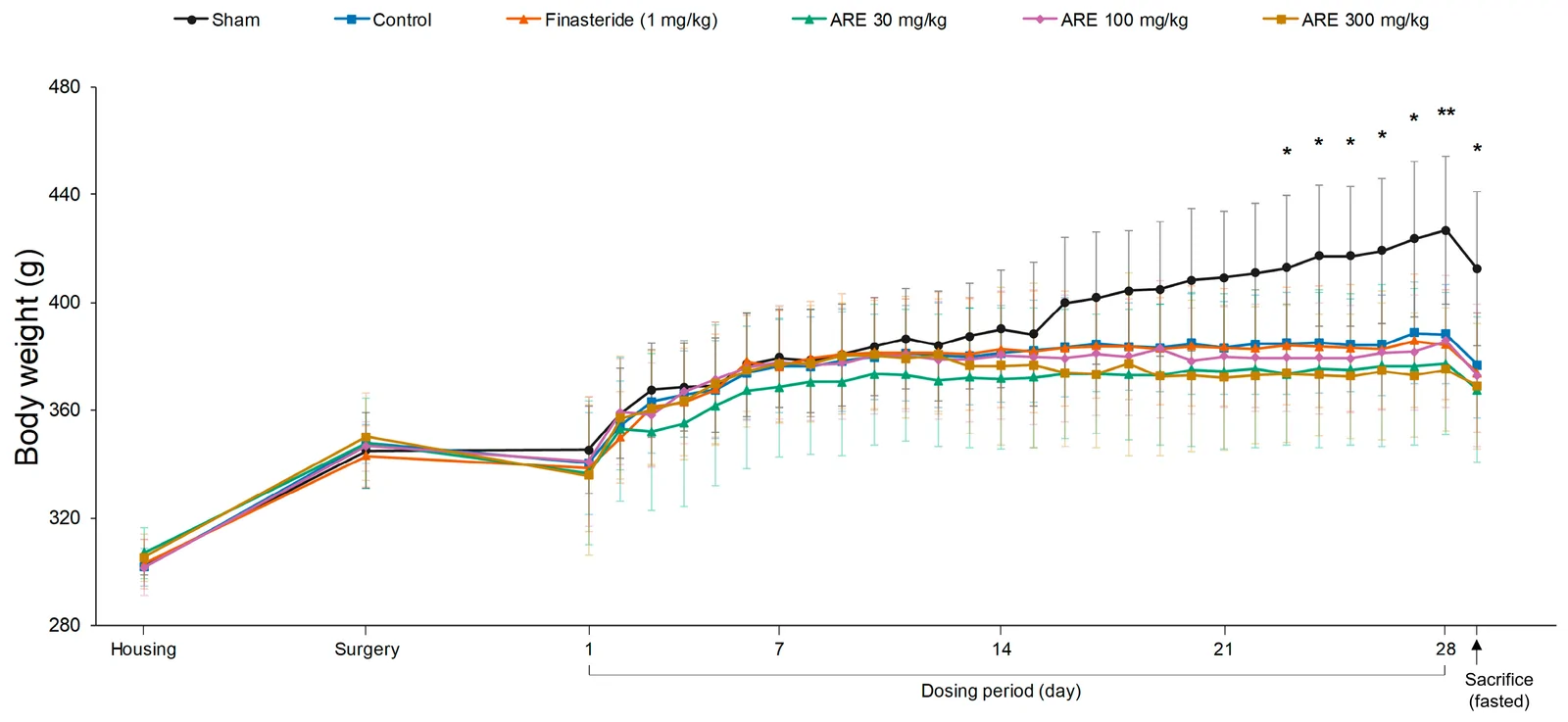
Body weight changes during the experimental period. Body weight was measured at housing, surgery (castration or sham surgery), daily during the 28-day dosing period, and at sacrifice after overnight fasting. Values are expressed as mean ± SD (*n* = 9 for sham and ARE groups; *n* = 10 for control and finasteride groups). * *p* < 0.05 and ** *p* < 0.01 vs. the control group at the same time point. ARE, Achyranthis Radix extract.

Body weights in the ARE and finasteride groups did not differ significantly from those in the control group at any time point (Figure 3). The sham group showed significantly higher body weights than the control group from Day 23 of the dosing period to sacrifice (*p* < 0.05 or *p* < 0.01). No abnormal clinical signs were observed in any group throughout the experimental period.

The fasted body weight of each animal at sacrifice was used to calculate its prostate index. Fasted body weights at sacrifice were 412.3 ± 28.7, 376.8 ± 19.4, 373.9 ± 22.3, 367.5 ± 26.8, 372.9 ± 26.3, and 368.8 ± 23.2 g in the sham, control, finasteride, ARE 30 mg/kg, ARE 100 mg/kg, and ARE 300 mg/kg groups, respectively.

### 3.3. Prostate Weight and Prostate Index

**Figure 4:**
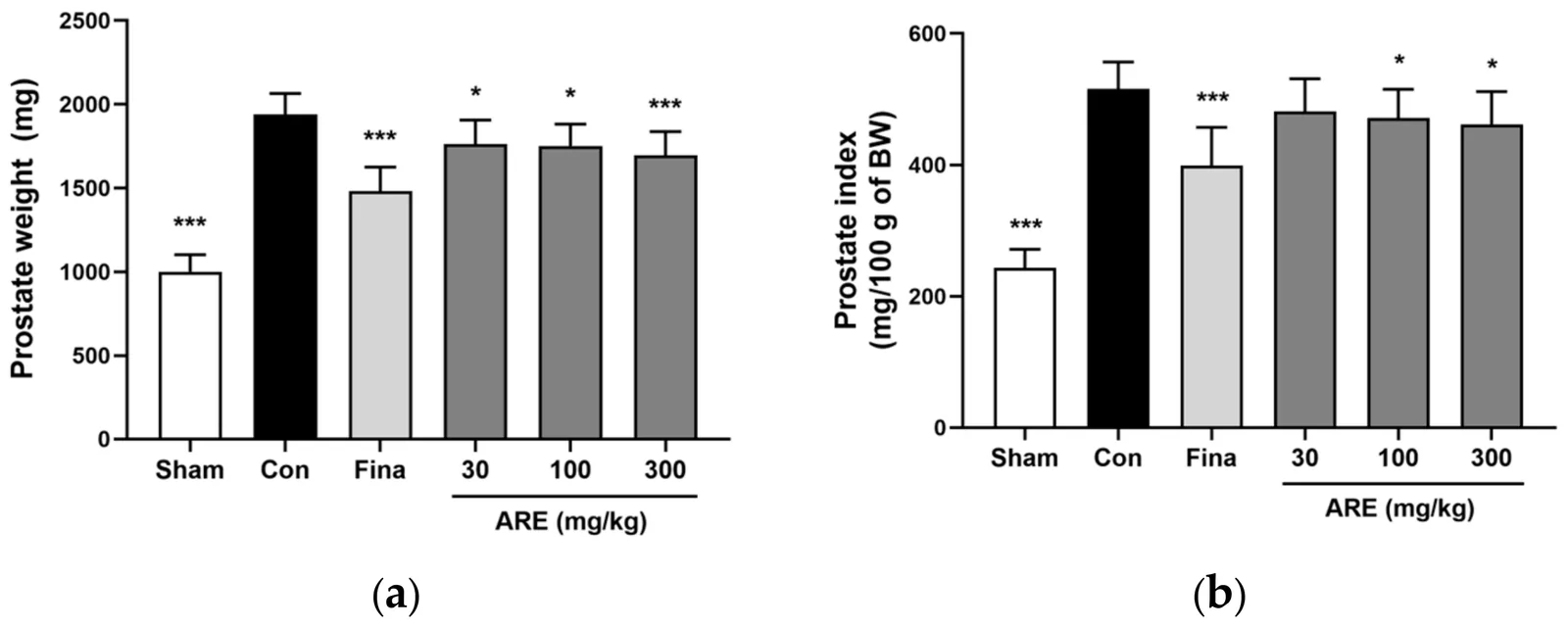
Prostate weight (**a**) and prostate index (**b**) following administration of Achyranthis Radix extract (ARE) in a rat model of benign prostatic hyperplasia induced by testosterone propionate. The prostate index represents prostate weight in milligrams per 100 g of body weight measured at sacrifice. All values are presented as the mean ± SD (*n* = 9 for sham and ARE groups; *n* = 10 for control and finasteride groups). * *p* < 0.05 and *** *p* < 0.001 vs. control group. BW, body weight; con, control; fina, finasteride 1 mg/kg.

Following 4 weeks of TP administration, mean prostate weight was 1939.8 ± 124.1 mg in the control group, approximately 1.9 times that in the sham group (999.6 ± 102.4 mg; *p* < 0.001; Figure 4a). Mean prostate weights were 1762.2 ± 144.4, 1751.8 ± 129.9, and 1695.2 ± 142.7 mg in the ARE 30, 100, and 300 mg/kg groups, respectively, with all three groups showing significantly lower values than the control group. For prostate index (prostate weight relative to body weight), the mean in the control group was approximately 2.1 times that in the sham group (516.0 ± 40.8 vs. 243.3 ± 28.3 mg/100 g body weight; *p* < 0.001; Figure 4b). ARE at 100 and 300 mg/kg significantly attenuated the TP-induced increase in prostate index by 16.3% and 20.0%, respectively (both *p* < 0.05). For prostate index, Hedges’ *g* values (95% confidence intervals) versus the control group were −0.73 (−1.61 to 0.18), −1.01 (−1.92 to −0.07), and −1.14 (−2.07 to −0.19) for ARE at 30, 100, and 300 mg/kg, respectively. Administration of the positive drug finasteride at 1 mg/kg significantly reduced the prostate weight and prostate index compared to the control group (both *p* < 0.001).

### 3.4. H&E Staining of Prostate Tissues

**Figure 5:**
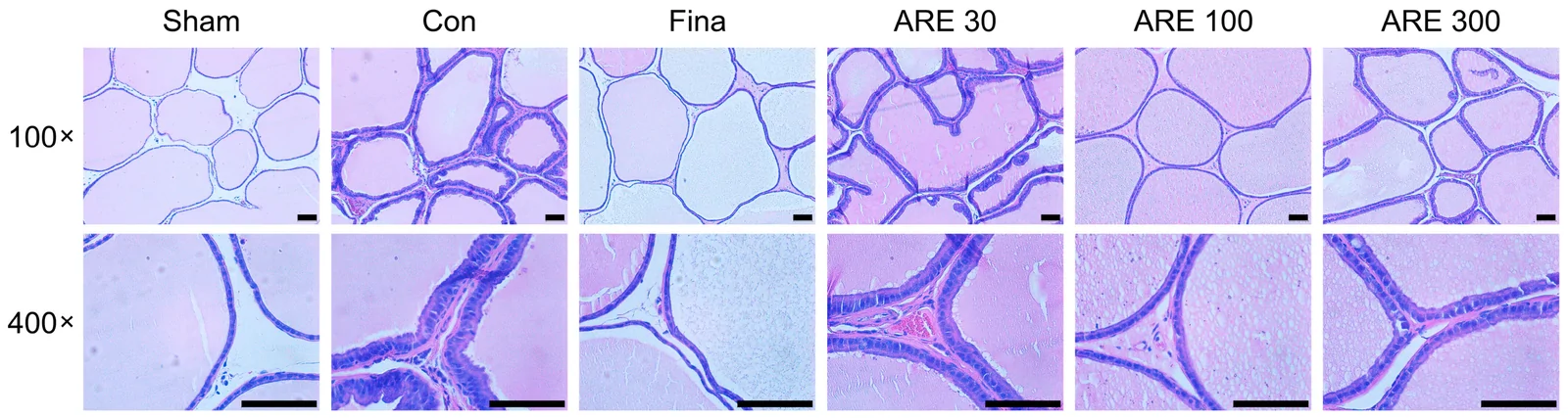
Representative micrographs of H&E-stained rat ventral prostate sections at 100× and 400× magnification. Achyranthis Radix extract (ARE) was administered at doses of 30, 100, and 300 mg/kg (indicated as ARE 30, 100, and 300, respectively). Scale bar = 100 μm at both magnifications. Fina, finasteride 1 mg/kg.

H&E-stained ventral prostate sections were examined to assess the anti-hyperplastic effects of ARE. Figure 5 presents representative micrographs from each group at 100× and 400× magnification. The sham group exhibited normal cell morphology, while the control group showed increased glandular hyperplasia and decreased luminal area. The administration of ARE at doses of 30, 100, and 300 mg/kg led to a reduction in glandular hyperplasia. Finasteride-treated rats exhibited cellular morphology similar to that observed in the sham group.

### 3.5. Histological Scores

The histological score, shown in Table 1, was evaluated based on histopathological changes in the ventral prostate at low-power (100×) and high-power (400×) magnifications. The sham group had a total score of 19.33 ± 7.63. In contrast, the control group’s total score was 48.90 ± 18.14, significantly higher than that of the sham group (*p* < 0.001). Injection of TP induced histological changes characteristic of prostatic hyperplasia, such as irregular acini, altered nuclear shape and size, an interrupted basement membrane, and piling up and budding out of epithelial cells. The administration of ARE and finasteride alleviated these pathological changes. Total histological scores were 26.11 ± 16.53, 29.67 ± 13.34, and 25.30 ± 15.56 in the ARE 100 mg/kg, ARE 300 mg/kg, and finasteride 1 mg/kg groups, respectively. All three groups had significantly lower scores than the control group.

### 3.6. Epithelial Thickness of the Prostate

**Figure 6:**
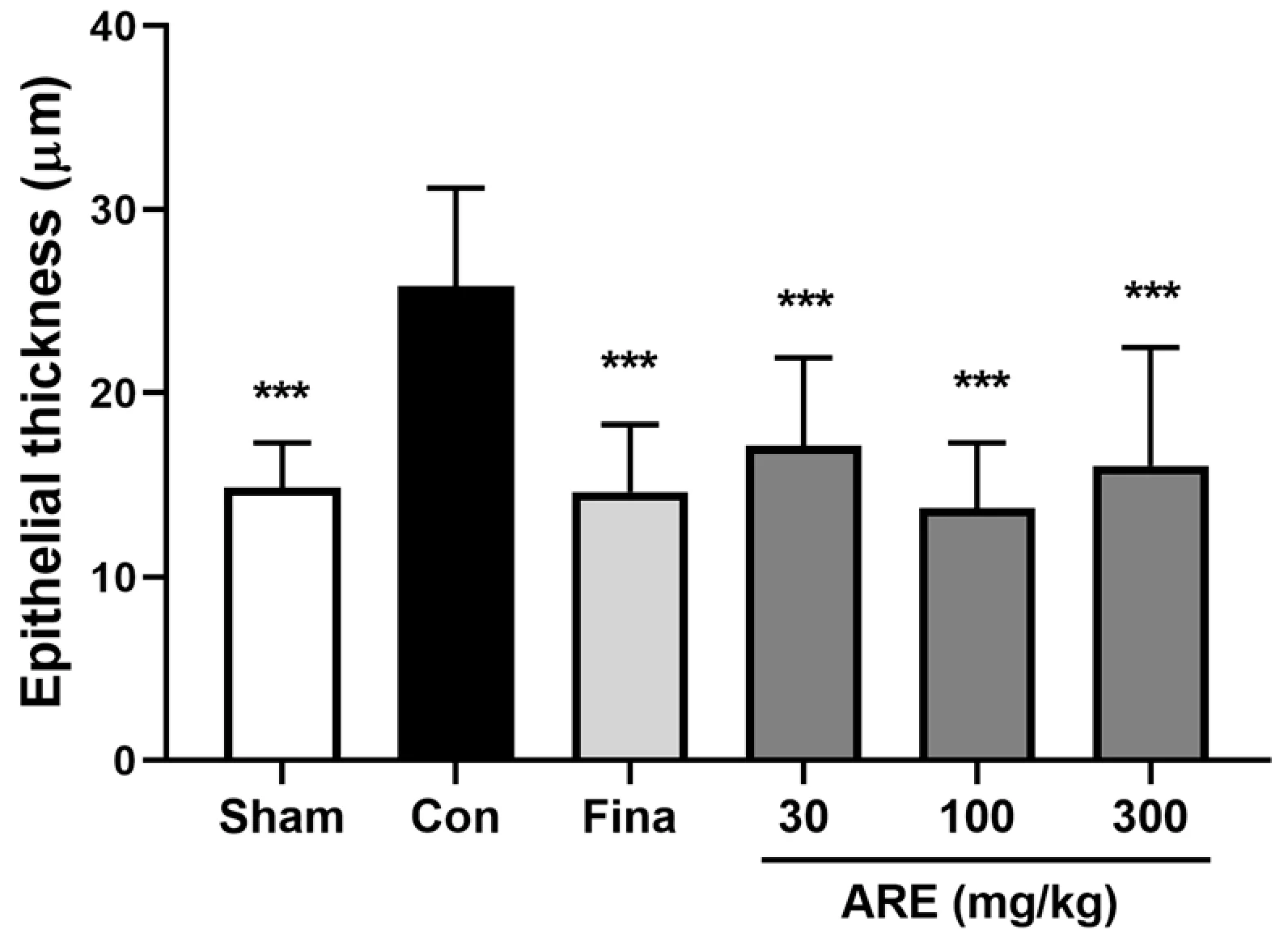
Effects of Achyranthis Radix extract (ARE) on the epithelial thickness of the prostate in rats injected with testosterone propionate. Data are expressed as the mean ± SD (*n* = 9 for sham and ARE groups; *n* = 10 for control and finasteride groups). *** *p* < 0.001 vs. control group. Con, control; fina, finasteride 1 mg/kg.

The epithelial thickness of the control group significantly increased compared to the sham group (25.8 ± 5.3 vs. 14.9 ± 2.4 µm, *p* < 0.001; Figure 6). Epithelial thickness was significantly lower in all ARE-treated groups (30, 100, and 300 mg/kg) and the finasteride group (1 mg/kg) than in the control group (all *p* < 0.001). The mean values were 17.2 ± 4.7 µm for the ARE 30 mg/kg group, 13.8 ± 3.6 µm for the ARE 100 mg/kg group, 16.1 ± 6.4 µm for the ARE 300 mg/kg group, and 14.6 ± 3.7 µm for the finasteride 1 mg/kg group.

### 3.7. Serum Concentrations of Testosterone and DHT

**Figure 7:**
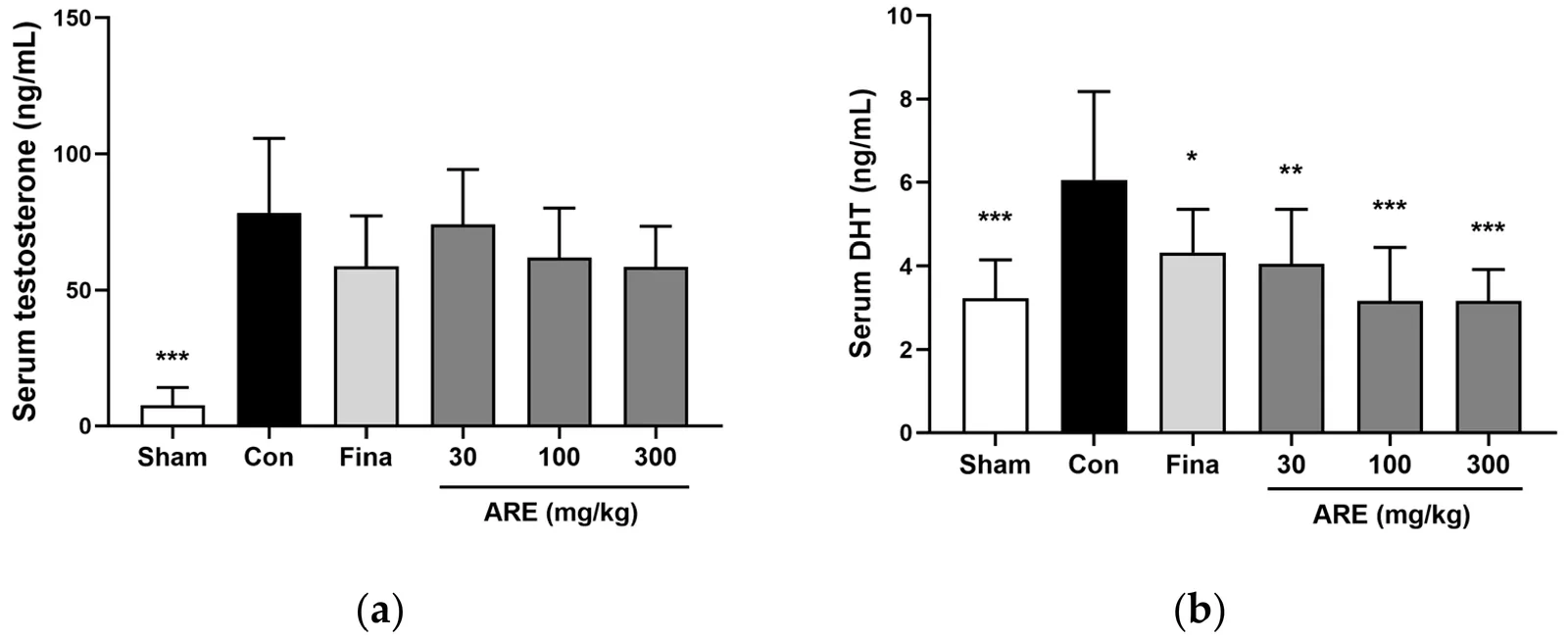
Serum testosterone (**a**) and dihydrotestosterone (DHT, (**b**)) concentrations following treatment with Achyranthis Radix extract (ARE) in a rat model of testosterone propionate-induced benign prostatic hyperplasia. All values are presented as the mean ± SD (*n* = 9 for sham and ARE groups; *n* = 10 for control and finasteride groups). * *p* < 0.05, ** *p* < 0.01, and *** *p* < 0.001 vs. control group. Con, control; fina, finasteride 1 mg/kg.

The control group had significantly higher serum testosterone concentrations than the sham group (Figure 7a). Testosterone concentrations were numerically lower in the ARE 100 and 300 mg/kg groups and the finasteride 1 mg/kg group than in the control group, but these differences were not statistically significant. 

Mean serum DHT concentration in the control group was approximately 1.9 times that in the sham group (6.1 ± 2.1 vs. 3.2 ± 0.9 ng/mL; *p* < 0.001; Figure 7b). All ARE-treated groups (30, 100, and 300 mg/kg) and the finasteride group (1 mg/kg) showed significantly lower serum DHT concentrations than the control group. The mean concentrations in the ARE 100 and 300 mg/kg groups were 3.2 ± 1.3 and 3.2 ± 0.8 ng/mL, respectively, both close to the sham mean.

### 3.8. Expression of AR and Apoptosis-Related Proteins in the Prostate

**Figure 8:**
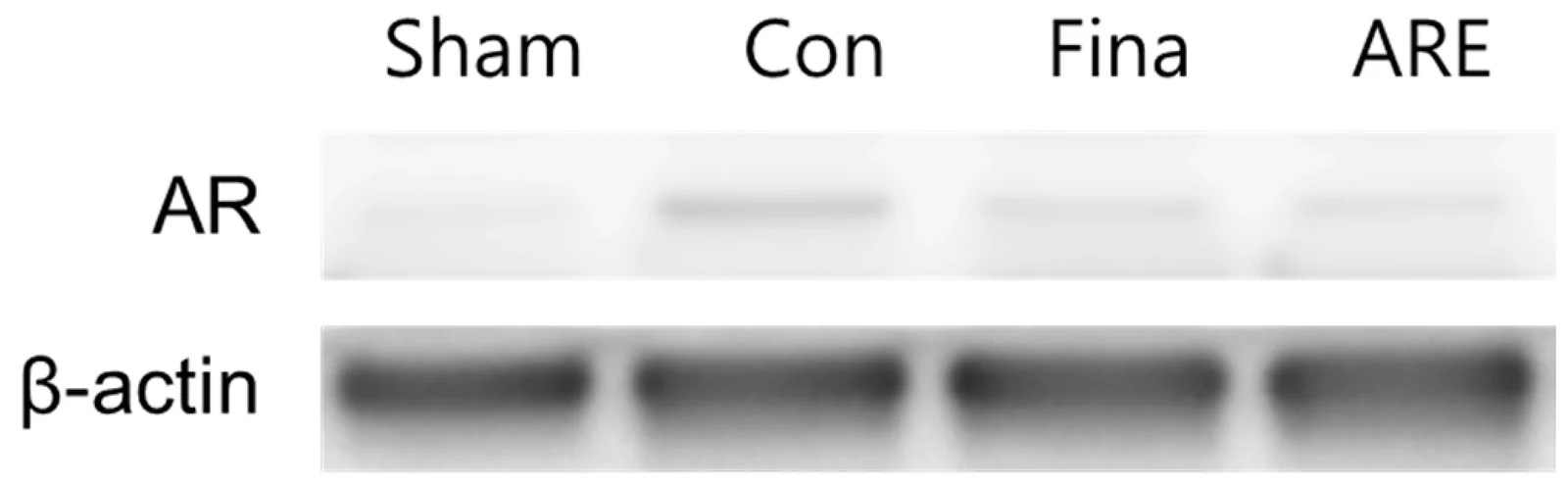

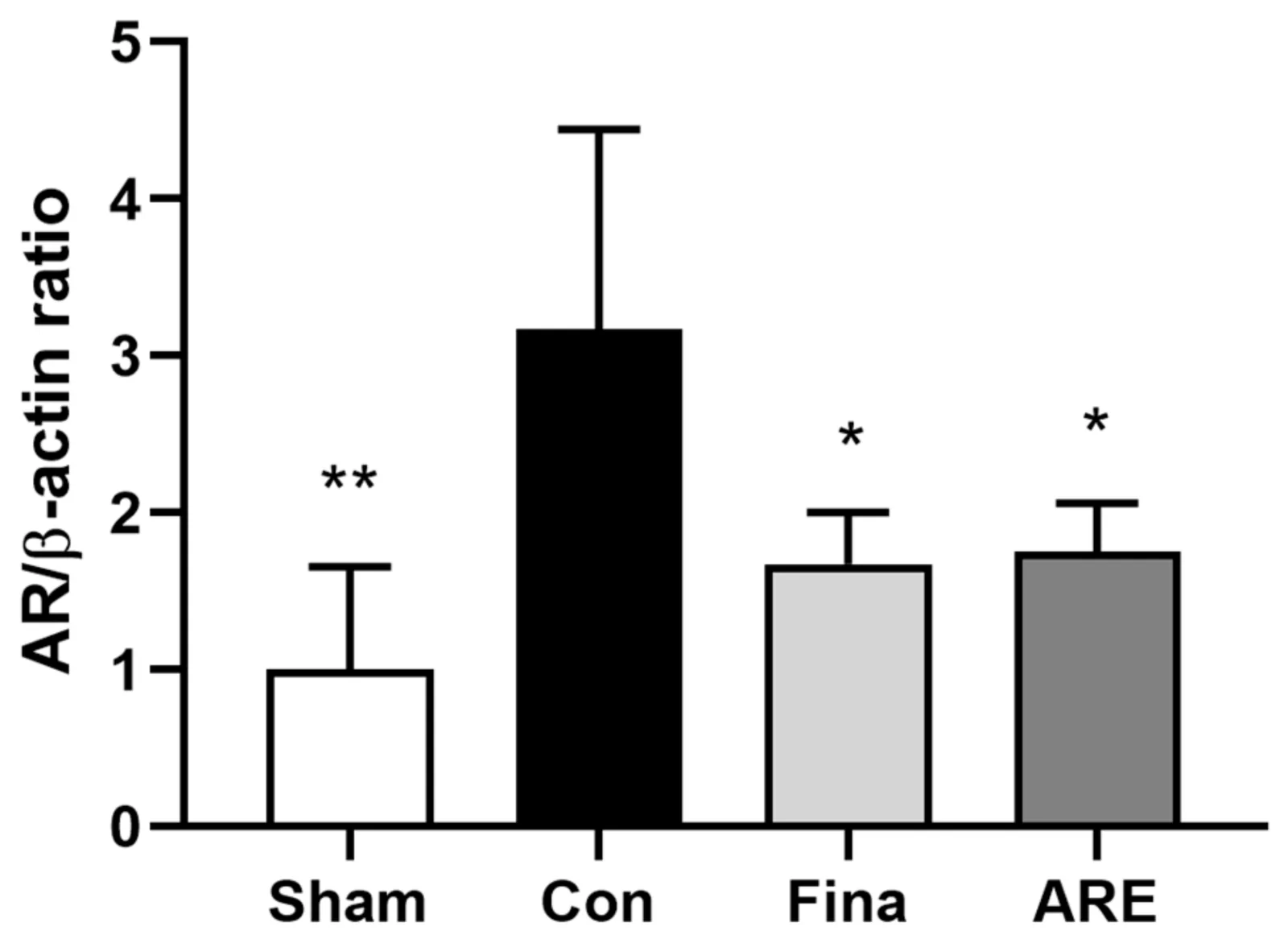
Androgen receptor (AR) protein expression in the ventral prostate following treatment with Achyranthis Radix extract (ARE) in testosterone propionate-treated rats. The treatment doses were 1 mg/kg for finasteride (Fina) and 300 mg/kg for ARE. Values are expressed as the mean ± SD (*n* = 4 biologically independent animals per group). * *p* < 0.05 and ** *p* < 0.01 vs. control group.

Following the finding that ARE decreased serum DHT levels, we next assessed the effect of ARE on AR protein expression in prostate tissue. The control group showed increased AR protein expression relative to the sham group, as depicted in Figure 8. This increase was significantly attenuated by ARE at 300 mg/kg and finasteride at 1 mg/kg.

**Figure 9:**
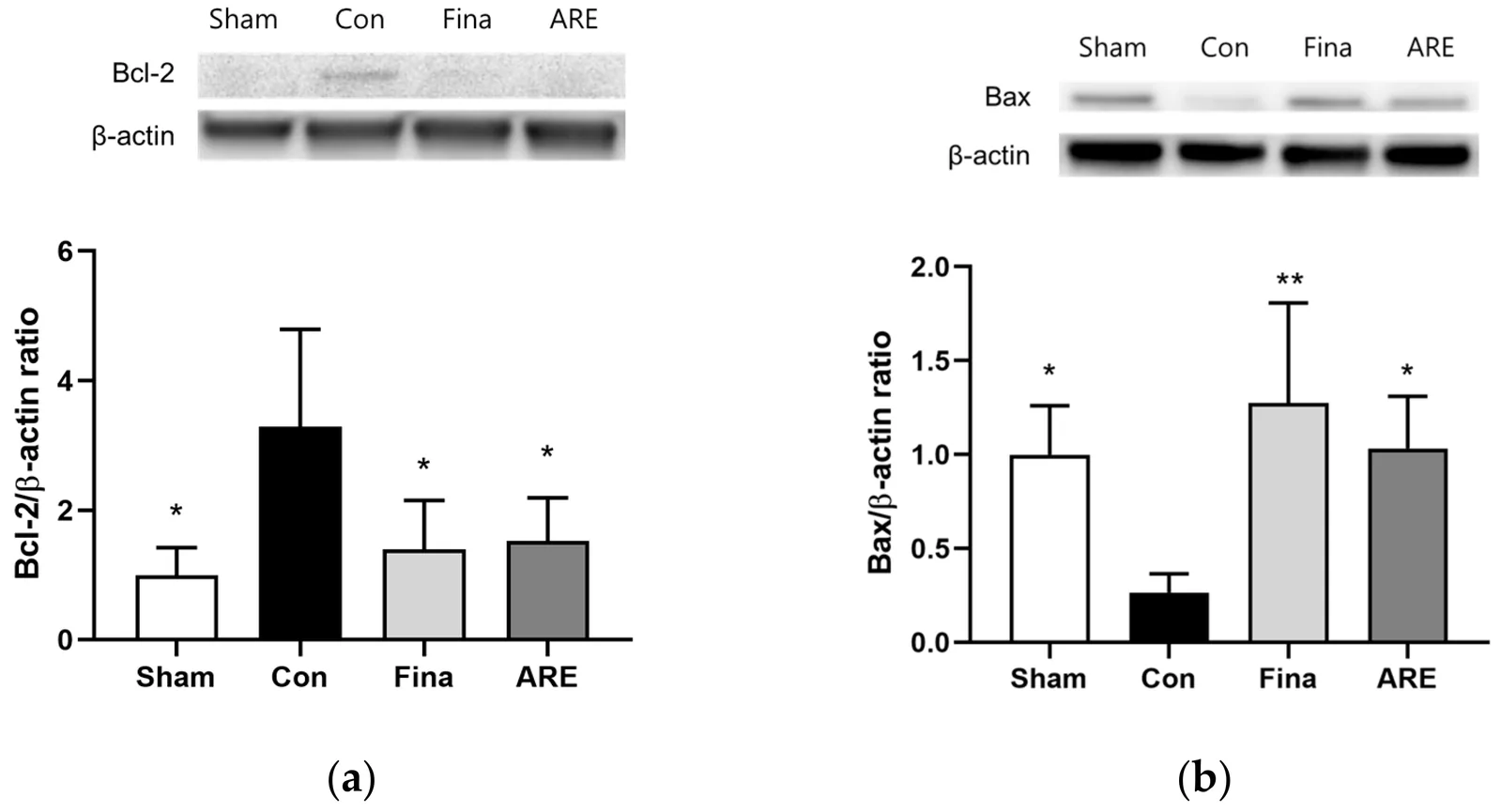
Bcl-2 (**a**) and Bax (**b**) protein expression in the ventral prostate following treatment with Achyranthis Radix extract (ARE) in testosterone propionate-treated rats. The treatment doses were 1 mg/kg for finasteride (Fina) and 300 mg/kg for ARE. Values are expressed as the mean ± SD (*n* = 4 biologically independent animals per group). * *p* < 0.05 and ** *p* < 0.01 vs. control group.

To assess the effects of ARE on apoptosis-related protein expression, the levels of Bcl-2 and Bax were examined in the ventral prostate. Compared to the control group, ARE at 300 mg/kg significantly decreased Bcl-2 expression and increased Bax expression, as shown in Figure 9. 

## 4. Discussion

This study explored the inhibitory effects of ARE on TP-induced prostatic hyperplasia in rats. The results demonstrated that oral treatment with ARE significantly suppressed the development of BPH, as indicated by decreased prostate index and mitigated histopathological alterations. Furthermore, ARE reduced serum DHT levels and altered the protein expression of AR and apoptosis-related biomarkers in the prostate.

The rat model of BPH induced by TP is a well-established model applied across various BPH research areas [36]. Administering TP in doses exceeding the physiological range to castrated rats induces prostatic hyperplasia, leading to an increase in both the absolute and relative weights of the prostate. In this model, castration is not necessary but, if performed, offers the advantage of ruling out the effects of endogenous testosterone [36]. In the present study, the injection of TP into castrated rats resulted in an approximately twofold increase in both prostate weight and prostate index compared to the sham group, confirming the successful induction of prostatic hyperplasia. ARE significantly suppressed both prostate weight and prostate index compared to the control rats, suggesting its inhibitory effects on prostatic hyperplasia.

ARE reduced the prostate index, with the greatest reduction observed at 300 mg/kg/day. Using body surface area-based conversion [30], the human-equivalent dose is calculated as 300 × (6/37) ≈ 48.6 mg/kg/day. For a 60-kg adult, this corresponds to approximately 2.9 g of extract/day (48.6 × 60/1000). Based on the extraction yield of 50.9%, the corresponding raw material dose is approximately 5.7 g/day (2.9/0.509). According to the Chinese Pharmacopoeia (2020 ed.), the traditional daily dosage of Achyranthis Radix (raw material) ranges from 5 to 12 g [28]. This estimated raw-material equivalent falls within the traditional dosage range. However, body surface area-based conversion does not account for interspecies differences in pharmacokinetics, and human pharmacokinetics and safety were not evaluated in this study. The clinical efficacy and safety of ARE at the corresponding human dose remain to be established.

BPH is typically characterized by the significant proliferation of epithelial and stromal cells, leading to enlargement of the gland [37]. In this study, prostate tissue from the control group displayed a cylindrical epithelial shape, with epithelial cells demonstrating piling up and budding out, indicative of epithelial proliferation [31,32]. Moreover, the interruptions and thickening of the basement membrane, a critical fibrous structure for maintaining tissue integrity, were pronounced. The basement membrane contributes to the maintenance of epithelial polarity and tissue homeostasis in the prostate [38]. Similar epithelial changes, including piling up of epithelial cells and increased epithelial thickness, have been reported in a testosterone-induced rat model of BPH [39]. Quantitative assessments revealed that ARE administration resulted in a reduction in epithelial thickness and a decrease in the overall histological score, marked by a significant reduction in both the interruption and thickening of the basement membrane. Together, these histopathological findings, alongside the observed reduction in prostate index attributable to ARE, suggest that ARE exerts inhibitory effects on the progression of BPH.

It is well established that androgen/AR signaling plays a pivotal role in the pathogenesis of BPH. Despite its heterogeneous etiology, the development of BPH in humans necessitates a combination of aging and testicular androgens [40]. DHT, produced from testosterone by the action of 5-alpha reductase, exhibits significantly increased affinity for androgen receptors, thereby enhancing its activity to be 4–5 times that of testosterone, making it a more potent androgen [41]. DHT plays a key permissive role in the development of BPH [42]. Upon the binding of DHT, AR translocates into the nucleus, stimulating the transcription and subsequent synthesis of growth factors that lead to the proliferation of prostate cells [7,41]. We found that ARE significantly reduced serum DHT concentrations and decreased prostatic AR protein expression. In prostate epithelial and stromal cell lines, DHT increased AR protein expression [43]. In rat ventral prostate, castration reduced AR protein content, whereas testosterone replacement restored it [44]. These experimental findings provide a biological basis for considering the reductions in serum DHT levels and prostatic AR protein expression observed in the present study as potentially related. These androgen-related changes may contribute to the anti-hyperplastic effects of ARE, although a causal relationship remains to be established.

The dysregulation of apoptosis has also been linked to BPH [45]. Epithelial atrophy and a transient increase in apoptosis have been observed in the prostates of men treated with finasteride [46]. To assess the effects of ARE on apoptosis-related protein expression, we analyzed the expression levels of Bcl-2 and Bax, two key proteins belonging to the Bcl-2 family that regulate apoptosis. Bax and Bcl-2 can form homodimers or heterodimers with each other, thereby influencing apoptotic regulation. An elevation in Bax is associated with enhanced apoptosis, whereas an increase in Bcl-2 levels suppresses apoptotic processes [47]. An increased Bax/Bcl-2 ratio has been associated with androgen withdrawal-induced apoptosis in the prostate [48,49]. Additional experimental evidence links androgen stimulation to changes in Bcl-2-family protein expression in prostate cells. In prostate epithelial and stromal cell lines, DHT increased Bcl-2 expression while decreasing Bax expression [43]. Our results showed that ARE decreased Bcl-2 expression while increasing Bax expression in the prostate. These findings provide a biological rationale for considering the observed changes in serum DHT levels and prostatic Bcl-2 and Bax protein expression as potentially related aspects of the regulation of prostate cell growth and survival. Further studies would help determine whether these changes are causally related and whether apoptosis is increased.

Increasing the ARE dose from 100 to 300 mg/kg did not result in clear additional reductions in prostate index, histological parameters, or serum DHT levels. This pattern may reflect a plateau in the response to ARE within this dose range. Nevertheless, with only three dose levels spanning a tenfold range (30–300 mg/kg) and variability in the measured responses, the present data cannot establish a plateau or exclude a smaller incremental benefit at the higher dose. Further dose-ranging studies would help clarify the relationship between dose and effect size.

Several limitations should be considered when interpreting these findings. The TP-induced BPH model in orchiectomized rats is widely used to evaluate agents targeting androgen-stimulated prostate growth under controlled experimental conditions [36]. However, it does not fully reproduce the gradual, age-related and multifactorial features of human BPH. Although spontaneous, age-associated BPH has been reported in chimpanzees and dogs [36], financial and ethical considerations limit the use of these animals in experimental research. Further studies are needed to establish the efficacy and safety of ARE in humans. In addition, the observed changes in serum DHT levels and prostatic AR, Bcl-2, and Bax expression do not establish 5α-reductase inhibition, functional suppression of AR signaling, or induction of apoptosis. Further mechanistic studies assessing 5α-reductase activity, AR transcriptional activity, and apoptosis using cleaved caspase-3 analysis or TUNEL staining would help clarify the mechanisms underlying the effects of ARE. Finally, phytochemical characterization of ARE was limited to quantification of β-ecdysterone as a quality control marker, and the constituents responsible for the observed effects remain to be identified.

The present findings provide preclinical evidence relevant to the traditional use of Achyranthis Radix in herbal prescriptions for BPH [24,25,26]. To the best of our knowledge, this is the first study to demonstrate the inhibitory effects of Achyranthis Radix as a single-herb extract on TP-induced prostatic hyperplasia in rats.

## 5. Conclusions

Taken together, our results suggest that ARE attenuated the development of prostate enlargement and associated histological changes in rats when administered concurrently with TP. Changes in serum androgen levels and androgen- and apoptosis-related protein expression in the prostate provide a basis for further mechanistic investigation. These findings support further evaluation of ARE as a potential option for BPH management.

## Figures and Tables

**Table 1:** Histological score.

Variable (Score Values)	Sham	Control	Fina	ARE 30	ARE 100	ARE 300
**Low-Power Magnification**						
*Luminal shape* regular (1), villous (3), papillary (4), cribriform (5)	1.22 (0.67) ^1^	1.80 (1.03)	1.20 (0.63)	1.89 (1.05)	1.78 (1.56)	1.44 (0.88)
*Acinar shape* tubular (1), branched (3), irregular (5)	1.00 (0.00)	2.20 (1.93)	1.40 (1.26)	1.44 (1.33)	1.44 (1.33)	1.89 (1.76)
*Interacinar space* large or moderate (1); back-to-back glands (5)	1.00 (0.00)	2.20 (1.93)	1.40 (1.26)	2.78 (2.11)	1.44 (1.33)	3.22 (2.11)
*Stroma* fine (1), abundant (3), fibrosis/severe smooth muscle hyperplasia (5)	2.33 (1.00)	2.80 (1.48)	2.20 (1.40)	2.11 (1.45)	2.11 (1.05)	1.44 (0.88)
**High-Power Magnification**						
*Epithelial shape* flattened (1), cuboidal (1), cylindrical (3), hexagonal (5)	1.44 (0.88)	2.00 (1.05)	1.60 (0.97)	1.67 (1.00)	1.22 (0.67)	1.44 (0.88)
*Number of layers* mono (1), oligo 2–4 (3), pluri, >5 (5)	1.44 (0.88)	2.20 (1.03)	1.20 (0.63)	1.67 (1.00)	1.22 (0.67)	1.44 (0.88)
If >1, then add focal (3), diffuse (5)	0.67 (1.32)	1.80 (1.55)	0.30 (0.95)	1.33 (1.58)	0.33 (1.00)	0.67 (1.32)
*Alignment* polar (1), apolar (3)	1.00 (0.00) *	2.00 (1.05)	1.20 (0.63)	1.67 (1.00)	1.22 (0.67)	1.44 (0.88)
If there is piling up of epithelial cells, add (3)	1.33 (1.58)	2.70 (0.95)	1.50 (1.58)	2.00 (1.50)	1.33 (1.58)	2.00 (1.50)
If there is budding out of epithelial cells into the stroma, add (5)	0.00 (0.00) **	3.50 (2.42)	2.00 (2.58)	2.22 (2.64)	2.78 (2.64)	1.11 (2.20)
If periacinar clusters of epithelial cells are found, add (3)	0.33 (1.00)	0.90 (1.45)	0.30 (0.95)	1.00 (1.50)	0.00 (0.00)	0.00 (0.00)
If isolated clusters of epithelial cells are found outside acini, add (5)	0.56 (1.67)	2.50 (2.64)	0.50 (1.58)	0.00 (0.00) *	0.56 (1.67)	1.11 (2.20)
*Lesion distribution* (for apolar or budding out cells, no lesion = 0): unilobar: isolated (2): multiple (6). Bilobar: isolated (4); multiple (8)	1.33 (2.00) *	4.80 (3.16)	2.00 (2.83)	3.11 (3.33)	1.78 (2.91)	2.22 (2.91)
*Nuclear shape* round, regular (1), irregular (5)	1.00 (0.00)	3.00 (2.11)	1.40 (1.26)	2.33 (2.00)	1.89 (1.76)	2.78 (2.11)
*Nuclear size* small (2), large (2), small and large in the same acinus (4)	2.00 (0.00) *	3.00 (1.05)	2.20 (0.63)	2.67 (1.00)	2.22 (0.67)	2.89 (1.05)
*Mitoses per field* absent (0); isolated, 1–2 (2); abundant, 3–5 (5); excessive, >5 (10)	0.22 (0.67) *	2.30 (2.06)	1.30 (1.64)	0.67 (1.00)	0.56 (1.67)	1.22 (1.72)
*Basement membrane* intact (1); interrupted (5)	1.44 (1.33) ***	5.00 (0.00)	2.20 (1.93) **	2.78 (2.11) *	2.33 (2.00) **	1.89 (1.76) ***
Thin (1); thick (5)	1.00 (0.00) ***	4.20 (1.69)	1.40 (1.26) ***	2.78 (2.11)	1.89 (1.76) **	1.44 (1.33) **
**Total score (arbitrary units)**	19.33 (7.63) ***	48.90 (18.14)	25.30 (15.56) **	34.11 (18.50)	26.11 (16.53) *	29.67 (13.34) *

Histological variables and score values are based on the scoring systems described in [31,32]. ^1^ Data are expressed as mean (SD). *n* = 9 for sham and ARE groups; *n* = 10 for control and finasteride groups. * *p* < 0.05, ** *p* < 0.01, and *** *p* < 0.001 vs. control group. ARE 30, 100, and 300 indicate Achyranthis Radix extract 30, 100, and 300 mg/kg, respectively; con, control; fina, finasteride 1 mg/kg.

## Data Availability

The datasets generated and analyzed during the current study are available from the corresponding author upon reasonable request.

## Abbreviations

The following abbreviations are used in this manuscript:

ARE	Achyranthis Radix extract
AR	Androgen receptor
BPH	Benign prostatic hyperplasia
DHT	Dihydrotestosterone
H&E	Hematoxylin–Eosin
HPLC	High-Performance Liquid Chromatography
LUTS	Lower urinary tract symptoms
TBST	Tris-buffered saline containing 0.1% Tween 20
TP	Testosterone propionate
